# Health literacy of common ocular diseases in Nepal

**DOI:** 10.1186/1471-2415-14-2

**Published:** 2014-01-08

**Authors:** Mohan Krishna Shrestha, Christina W Guo, Nhukesh Maharjan, Reeta Gurung, Sanduk Ruit

**Affiliations:** 1 Tilganga Institute of Ophthalmology, PO Box 561, Kathmandu, Nepal; 2 University of Melbourne, Parkville, Victoria 3052, Australia

**Keywords:** Cataract, Diabetic retinopathy, Glaucoma, Health literacy, Nepal, Night blindness, Trachoma

## Abstract

**Background:**

Poor health literacy is often a key cause of lack of or delayed uptake of health care services. The aim of this study was to assess the health literacy of common ocular diseases, namely cataract, glaucoma, night blindness, trachoma and diabetic retinopathy in Nepal.

**Methods:**

A cross sectional study of 1741 participants randomly selected from non-triaged attendants in the outpatient queue at Tilganga Institute of Ophthalmology, a semi urban general population of Bhaktapur district of Kathmandu Valley and patients attending rural outreach clinics. Participants responded to trained enumerators using verbally administered, semi structured questionnaires on their awareness and knowledge of cataract, glaucoma, diabetic retinopathy, night blindness, and trachoma.

**Results:**

The awareness of cataract across the entire sample was 49.6%, night blindness was 48.3%, diabetic retinopathy was 29%, glaucoma was 21.3% and trachoma was 6.1%. Patients presenting to rural outreach clinics had poorer awareness of cataract, glaucoma, diabetic retinopathy, night blindness and trachoma compared to those from a semi-urban community and an urban eye hospital (p<0.05), Old age was directly associated with poorer awareness of cataract, glaucoma, night blindness, trachoma and diabetic retinopathy (p<0.05). Female gender was associated with lower awareness of cataract, glaucoma, night blindness and trachoma (p<0.05). Literacy was associated with greater awareness of cataract, glaucoma, diabetic retinopathy, night blindness and trachoma (p<0.05). Higher education was significantly associated with greater awareness of cataract, night blindness and trachoma (p<0.05). Multivariate analysis found that the awareness of common ocular diseases was significantly associated with level of education (p<0.05). Similarly, awareness of cataract, glaucoma, trachoma and night blindness was associated with female gender (p<0.05) whereas awareness of cataract, night blindness, trachoma and diabetic retinopathy was associated with age (p<0.05) but the awareness glaucoma and diabetic retinopathy was associated with camps.

**Conclusions:**

Low awareness of common ocular conditions is associated with factors such as female gender, old age, lower levels of education and rural habitation. A would be successful health promotion programs should specifically target health determinants to promote health literacy and to ensure timely utilization of eye care services.

## Background

In 1981, the Nepal Blindness Survey provided the first epidemiological information on the prevalence of blindness in Nepal. The prevalence of blindness in Nepal was estimated to be 0.84% and was among the poorer in the world [1]. The Rapid Assessment of Avoidable Blindness Survey in 2012 of Nepal found that the prevalence of blindness was 0.35% [2]. Despite the expansion of eye health services, preventable and correctable ocular conditions such as cataract remain leading causes of blindness in Nepal [3-7].

It has long been recognized that in both developing and developed countries, poor health literacy is a key cause of lack of or delayed uptake of health care services, lack of compliance to treatment and poor follow-up [8-12]. Health literacy is defined as the ability of an individual to access, understand and use information to promote and maintain good health [13]. It has been suggested that poor ocular health literacy is a key contributor to the dichotomy that exists between disease prevalence and service uptake [5,14-16]. In an Indian study, it was found that awareness and knowledge of common ocular conditions, such as cataract, glaucoma, night blindness, trachoma and diabetic retinopathy was very poor [9]. Having heard of the disease was defined as “awareness” and having some understanding of the basic etiology and symptoms of the disease was defined as “knowledge”. At present, no comprehensive study on the awareness and knowledge of common ocular conditions exists for the Nepalese population.

Health education and promotion is the one of the most powerful tools to increase the health literacy, as a public health goal, in different settings or localities [17-19]. As Nepal is a developing country, eye health system here is more focused on the curative services rather than promotive and preventive services. It is the best time to assess and explore the situation of the awareness and knowledge of common eye diseases among different groups of people to help the eye health authorities to make and implement a good plan which is both cost effective and efficient in different social and geographic settings in Nepal.

The aim of this study was to investigate the awareness and knowledge of common causes of blindness, namely: cataract, glaucoma, diabetic retinopathy, night blindness and trachoma in a semi-urban community of Kathmandu Valley; patients presenting to an urban eye hospital and; patients presenting to rural outreach clinics (camps). Since health literacy itself is a multi- factorial entity, the study also investigated the relationship between lack of awareness and other risk factors of poor ocular health such as gender, location of habitation, level of education, literacy and age.

## Methods

The cross sectional study was conducted to determine the awareness and knowledge of cataract, glaucoma, night blindness, trachoma and diabetic retinopathy in 1741 respondents over a three month period (March through May in 2009). The sample size was determined by the assumed status of awareness and knowledge of the diseases. Since the status of awareness and knowledge of these conditions are unknown in Nepal, the status of awareness and knowledge figures were derived from studies in India [9]. Night blindness and trachoma were of the lowest awareness and knowledge thus the assumed lowest status of awareness was 20% in all diseases with allowable error of 20%. Then the minimum sample size was 400 in each group. The study ultimately recruited 1741 participants.

Within each location, respondents were randomly selected including 893 respondents from the general semi-urban population of the Bhaktapur district of Kathmandu Valley, 413 respondents from the outpatient queue of an urban eye hospital (Tilganga Institute of Ophthalmology) and 431 respondents presenting to rural outreach clinics. The three groups were selected because they provided respondents from both rural and urban communities. They also represent the groups to which new health promotion measures could be immediately implemented. As free and accessible services are infrequent in rural communities, it is common for many patients who are free of disease symptoms to present themselves at rural outreach clinics. Thus, the sample of patients from four rural outreach clinics in Kathmandu Valley provides a representative sample of the rural community in general. Respondents aged 18 and above years were selected by two processes: (1) the process of simple random sampling using their registration number if they were at the hospital outpatient clinic and rural outreach clinic, or (2) the use of the latest community member list provided by the local government for the semi urban community from the Bhaktapur district of Kathmandu Valley. Questionnaires on awareness and knowledge of cataract, glaucoma, night blindness, trachoma and diabetic retinopathy were designed in English by the Research Department at Tilganga Institute of Ophthalmology, and translated into Nepali using a pragmatic approach to adapt to the target population. All questionnaires were verbally administered by field enumerators who spoke local language and had an understanding of the eye diseases of interest. They were trained and supervised on site by investigators from the research department of Tilganga Institute of Ophthalmology. The same enumerators and investigators were involved at all sites. The pretesting of questionnaires was conducted by enumerators on a similar semi-urban community from a different district of Kathmandu Valley, as well patients from both urban and rural backgrounds at the institute. Respondents demonstrated awareness if they had heard of the eye disease in question. All questions about knowledge were semi- structured. Respondents were asked to “tell” what threat the eye disease posed. The questionnaire contained a list of the possible responses, and the field enumerators marked against the response that most closely corresponded to the respondent’s answer.

Collected data was reviewed immediately on site by the enumerators, then centrally at the Tilganga Institute of Ophthalmology. The data was collated and entered into Microsoft Excel 2007 at the Research Department of Tilganga Institute of Ophthalmology. The data were analyzed using Statistical Package for Social Science (SPSS Inc., Chicago, IL, USA) version 11.5. The bivariate (Spearman’s Rho test, chi square test and odds ratio) and multivariate analysis was used to determine the relationship between age, gender, rural habitation, literacy and level of education with awareness. P values less than 0.05 were considered statistically significant.

The Institutional Review Committee of Tilganga Institute of Ophthalmology reviewed and gave approval to conduct research in the line with the Helsinki Declaration of Medical Ethics.

## Results

### Demographic information

One thousand seven hundred and forty one participants were included in this study. Eight hundred and ninety three (51.29%) were from the semi-urban communities in Bhaktapur district in Kathmandu Valley, 431 (24.76%) were from the outpatient queue at the Tilganga Institute of Ophthalmology, an urban eye hospital, and 417 (23.95%) were selected from rural outreach clinics. Their age ranged from 16 to 89 years and the mean age was 41.5 (SD 17.7) years. The male to female ratio was 1.2 (54.6% male and 45.4% female). Thirty three point four percent of respondents were illiterate, defined as no formal schooling (Table 1).

**Table 1:** Summary of demographic of respondents (n = 1741)

**Description**	**Total**	**Semi-urban general**	**Urban hospital**	**Rural outreach**
	**Number (%)**	**Number (%)**	**Number (%)**	**Number (%)**
**Location**				
Semi-urban general population	893(51.3)			
Urban hospital-outpatient clinic	431(24.8)			
Rural outreach clinics	417(24.0)			
**Age**				
<20	142(8.2)	56(6.3)	56(13.0)	30(7.2)
20-29	411(23.6)	196(21.9)	173(40.1)	42(10.1)
30-39	323(18.6)	175(19.6)	100(23.2)	48(11.5)
40-49	284(16.3)	172(19.3)	51(11.8)	61(14.6)
50-59	224(12.9)	121(13.5)	33(7.7)	70(16.8)
60-69	197(11.3)	97(10.9)	16(3.7)	84(20.1)
≥70	160(9.2)	76(8.5)	2(0.5)	82(19.7)
**Gender**				
Male	950(54.6)	448(50.2)	92(21.3)	251(60.2)
Female	791(45.4)	445(49.8)	339(78.7)	166(39.8)
**Level of education**				
No education	582(33.4)	315(35.3)	41(9.5)	226(54.2)
Less than primary	239(13.7)	138(51.5)	37(8.6)	64(15.3)
Primary	189(10.9)	92(10.3)	64(14.8)	33(7.9)
Secondary	341(19.6)	144(16.1)	135(31.3)	62(14.9)
Higher secondary	390(22.4)	204(22.8)	154(35.7)	32(7.7)
**Literacy rate**				
Illiterate	582(33.4)	315(35.3)	41(9.5)	226(54.2)
Literate	1159(66.6)	578(64.7)	390(90.5)	191(45.8)

### Awareness of common ocular conditions

Overall awareness was 49.6% (863) for cataract, 21.3% (370) for glaucoma, 48.3% (841) for night blindness, 6.1% (106) for trachoma and 29% (504) for diabetic retinopathy (Table 2 Part A).

**Table 2:** Association of awareness of cataract, glaucoma, night blindness, trachoma, diabetic retinopathy in relation to age, sex, education, literacy and location (n = 1741)

**Variables**	**Total number**	** Awareness**				
		**Cataract**	**Glaucoma**	**Night blindness**	**Trachoma**	**Diabetic retinopathy**
**Part A**						
Grand Total	1741	863(49.6)	370(21.3)	841(48.3)	1 06(6.1)	504(29.0)
**Part B: Location**^**a**^						
Semi-urban general population	893	484(54.2)	256(28.7)	473(52.9)	65(7.3)	326(36.5)
Urban hospital-outpatient clinic	431	237(54.9)	78(18.1)	252(58.5)	36(8.4)	112(25.9)
Rural outreach clinics (camps)	417	142(34.1)	36(8.6)	116(27.8)	5(1.2)	66(15.8)
**Part C: Age group(years)**^**b**^						
<20	142	84(59.2)	31(21.8)	105(73.9)	8(5.6)	52(36.6)
20-29	411	253(61.6)	112(27.3)	271(65.9)	41(10.0)	155(37.7)
30-39	323	175(54.2)	79(24.5)	157(48.6)	24(7.4)	108(33.4)
40-49	284	136(47.9)	68(23.9)	137(48.2)	22(7.7)	83(29.2)
50-59	224	91(40.6)	42(18.8)	83(37.1)	7(3.1)	50(22.3)
60-69	197	*63(32.0)*	21(10.7)	51(25.9)	2(1.0)	34(17.3)
≥70	160	61(38.1)	17(10.6)	37(23.1)	2(1.3)	22(13.8)
**Part D: Gender**^**c**^						
Male	950	532(56)	225(23.7)	514(54.1)	70(7.4)	290(30.5)
Female	791	331(41.8)	145(18.3)	327(41.3)	36(4.6)	214(27.1)
**Part E: Education**^**d**^						
No education	582	165(28.4)	65(11.2)	141(24.2)	11(1.9)	88(15.1)
Less than primary	239	118(49.4)	47(19.7)	101(42.3)	9(3.8)	69(28.9)
Primary	189	84(44.4)	35(18.5)	73(38.6)	10(5.3)	35(18.5)
Secondary	341	193(56.6)	67(19.6)	190(55.7)	18(5.3)	95(27.9)
Higher secondary	390	303(77.7)	156(40.0)	336(86.2)	58(14.9)	217(55.6)
**Literacy**^**e**^						
Literate	582	165(28.4)	65(11.2)	141(24.2)	11(1.9)	88(15.1)
Illiterate	1159	698(60.2)	305(26.3)	700(60.4)	95(8.2)	416(35.9)

^a^p<0.001 for cataract, glaucoma, night blindness, trachoma and diabetic retinopathy, Pearson chi-square test.

^b^p = 0.005 for cataract, p<0.001 for glaucoma, night blindness and diabetic retinopathy and P = 0.019 for trachoma, Spearman’s rho test.

^c^p<0.001 for cataract and night blindness, p = 0.007 for glaucoma, p = 0.014 for trachoma, and p = 0.112 for diabetic retinopathy, Pearson chi-square test.

^d^p=0.037 for cataract and night blindness, p = 0.188 for glaucoma and diabetic retinopathy, and p = 0.005 for trachoma, Spearman’s rho test.

^e^p<0.001 for cataract, glaucoma, night blindness, trachoma and diabetic retinopathy, Pearson chi-square test.

### Age

Old age was significantly associated with poorer awareness of cataract (Spearman’s Rho correlation coefficient (r) =0.94, p = 0.005), glaucoma (r = 1.00, p<0.001), night blindness (r = 1.00, p<0.001), trachoma (r = 0.89, p = 0.019) and diabetic retinopathy (r = 1.00, p<0.001) (Table 2, part C).

### Gender

Females had significantly lower awareness of cataract (odds ratio = 1.77 (95% confidence interval (CI) = 1.46-2.14, p < 0.001), glaucoma (odds ratio = 1.38 (95% CI = 1.09-1.75), p = 0.007), night blindness (odds ratio = 1.67 (95% CI = 1.38-2.02), p<0.001), trachoma (odds ratio = 1.67 (95% CI = 1.10-2.52), p = 0.014). The difference in awareness for diabetic retinopathy between males and females was not significant (odds ratio = 1.19 (95% CI = 0.96-1.46), p = 0.11) (Table 2, Part D).

### Location of participants

Patients presenting at rural outreach clinics had significantly poorer awareness of cataract (odds ratio = 2.32 (95% CI = 1.84-2.91), p < 0.001), glaucoma (odds ratio = 3.57 (95% CI = 2.48-5.14), p< 0.001), night blindness (odds ratio = 3.14 (95% CI = 2.47-3.99), p< 0.001), trachoma (odds ratio = 6.80 (95% CI = 2.75-16.82), p< 0.001) and diabetic retinopathy (odds ratio = 2.63 (95% CI = 1.97-3.50), p<0.001) compared to patients presenting to urban outpatient clinic at Tilganga Institute of Ophthalmology and the general semi-urban population of Kathmandu valley (Table 2, part B).

### Awareness and level of education

Illiterate participants (33.43%) had significantly poorer awareness of all ocular conditions investigated including cataract (odds ratio = 3.83 (95 CI = 3.09-4.75), p< 0.001), glaucoma (odds ratio = 2.84 (95% CI = 2.13-3.79), p<0.001), night blindness (odds ratio = 4.78 (95% CI = 3.82-5.96), p < 0.001), trachoma (odds ratio = 4.64 (95% CI = 2.46-8.72), p<0.001) and diabetic retinopathy (odds ratio = 3.14 (95% CI = 2.43-4.06), p< 0.001).

The level of education of participants and their awareness of the conditions was also investigated. The levels of education were divided into three categories: illiterate, those who have basic literacy but less than primary school education, those whose highest level of education was primary, lower secondary or higher secondary or tertiary.

Higher level of education was significantly associated with greater awareness of cataract (r = 0.90, p = 0.037), night blindness (r = 0.90, p = 0.037) and trachoma (r = 0.98, p = 0.005). However, the relationship between higher education and awareness of diabetic retinopathy (r = 0.70, p = 0.188) and glaucoma (r = 0.70, p = 0.188) were not significant (Table 2, Part E).

Multiple correlations showed that the awareness on female gender is significantly associated with cataract (r = −0.128, p = 0.000), glaucoma (r = −0.065, p = 0.008), trachoma (r = −0.060, p = 0.012) and night blindness (r = −0.115, p = 0.000) except diabetic retinopathy (r = −0.023, p = 0.198). The awareness is significantly associated with cataract (r = −0.142, p = 0.000), trachoma (r = −0.053, p = 0.025), night blindness (r = −0.23, p = 0.000) and diabetic retinopathy (r = −0.109, p = 0.000) expect glaucoma (r = −0.042, p = 0.060) by ageing of population. The awareness is significantly associated with cataract (r = 0.352, p = 0.000), glaucoma (r = 0.216, p = 0.000), trachoma (r = 0.155, p = 0.000), night blindness (r = 0.420, p = 0.000) and diabetic retinopathy (r = 0.265, p = 0.000) by higher education level. Whereas the awareness of common ocular diseases is significantly associated with glaucoma (r = −0.151, p = 0.000) and diabetic retinopathy (r = −0.142, p = 0.000) with rural outreach clinics (camps) but there is no association with cataract (r = −0.028, p = 0.147), trachoma (r = −0.014, p = 0.304) and night blindness (r = 0.008, p = 0.379) (Table 3).

**Table 3:** Association of awareness of cataract, glaucoma, night blindness, trachoma, diabetic retinopathy in relation to age, sex, education, literacy and location by multiple correlation analysis

**Variables**	**Cataract**		**Glaucoma**		**Trachoma**		**Night blindness**		**Diabetic retinopathy**	
	**r**	**p value**	**r**	**p value**	**r**	**p value**	**r**	**p value**	**r**	**p value**
Gender	−0.128	0.000	−0.065	0.008	−0.060	0.012	−0.115	0.000	−0.023	0.198
Age	−0.142	0.000	−0.042	0.060	−0.053	0.025	−0.230	0.000	−0.109	0.000
Education	0.352	0.000	0.216	0.000	0.155	0.000	0.420	0.000	0.265	0.000
Location	−0.028	0.147	−0.151	0.000	−0.014	0.304	0.008	0.379	−0.142	0.000

### Source of information

The survey also investigated the common sources of information for awareness and knowledge of common ocular conditions. The Television and print media such as magazines, posters and newspapers, were the source of information in 52% of cases. Twenty four point nine percent of respondents derived their awareness and knowledge of common ocular conditions from family, relatives and friends who suffer from the condition, while 9.4% respondents derived their information from family, relatives and friends who do not suffer from the conditions (9.4%). Health care workers were the source of awareness in 3.3% and outreach eye clinics were the source of information in 3.1% of cases (Table 4).

**Table 4:** Source of information for common ocular problems

**Description**	**Cataract**		**Glaucoma**		**Night blindness**		**Trachoma**		**Diabetic retinopathy**		**Total**	
	N*	% **	N*	% **	N*	% **	N*	% **	N*	% **	N*	% **
Television, print media	395	47.9	193	52.2	486	58.8	74	69.8	220	43.7	1368	52
Family/friend/relative who suffer from condition	249	30.2	82	22.2	140	16.9	9	8.5	174	34.5	654	24.9
Family/friend/relative who do not suffer from condition	73	8.9	34	9.2	77	9.3	9	8.5	54	10.7	247	9.4
Eye camp	28	3.4	17	4.6	25	3	1	0.9	10	2	81	3.1
Health care workers	36	4.4	21	5.7	10	1.2	2	1.9	17	3.4	86	3.3
Others	43	5.2	23	6.2	89	10.8	11	10.4	29	5.8	195	7.4
Total	824	100	370	100	827	100	106	100	504	100	2631	100

N* = Number, %** = Percent.

## Discussion

This study identified a number of factors associated with lower awareness of common ocular conditions in Nepal, where blindness due to treatable conditions remains high despite increasing efforts to improve eye health. The factors examined were age, gender, literacy, level of education and location of habitation.

Cataract is the principal cause of blindness in Nepal, followed by glaucoma and diabetic retinopathy and trachoma [20-22]. The awareness of ocular conditions correlated with the prevalence of these conditions with the exception of night blindness. The high level of awareness and low prevalence of night blindness is directly related to the Nepal National Vitamin A Program which is a six month yearly campaign that delivers high dose vitamin A to 6 to 60 months old children in 42 of the 75 districts in Nepal [23].

In Nepal, 69% of blind people are females [1]. However, there is not a majority of females to males in the Nepalese population. Also, this disparity is not accounted for by longer female life expectancy (0.88 males to 1 female in those over the age of 65 years) [24]. Females account for 63.8% of cataract blindness because they do not receive treatment at the same rate as men [1]. It is estimated that cataract blindness would be reduced by 12.5% in developing countries if women received cataract surgery at the same rate as men [25]. Given the significant difference in level of awareness of common ocular conditions between males and females, it is likely that low level of awareness plays an important role. Potential causes for this include the fact that only 24% of females in Nepal are literate as opposed to 52% of males. Females are also more likely to live in a rural location and have lower income [24,26,27]. All these factors reduce access to health education, and ultimately, affect health seeking behaviour. Any programs aimed at improving health literacy must specifically address gender specific barriers. Programs aimed at improving other determinants such as poor literacy should also be gender-sensitive.

The highest rate of blindness in Nepal is for those who are over 70 years of age where prevalence is over 10.6% [28]. Even in developed countries, the risk of blindness is ten times higher in those over 65 than for younger individuals [29,30]. There are many causes for the under-utilization of services in the elderly. For example, the elderly are more likely to associate decline in vision with natural aging, less likely to present for surveillance and monitoring, more conservative in the uptake of procedures, less compliant with medications and follow-up [31-33]. This may be related to the fact that literacy rates decline with age in both males and females, and urban and rural communities. For example, literacy amongst 10–14 year olds is 86% for males and 83% for females compared to the literacy rate of 34% for older males and 3% for older females [34]. The Elderly are also more likely to live in rural areas [35]. Hence, there is a need for culturally secure educational programs targeted at the elderly.

Eighty percent of the Nepalese population and ninety percent of country’s blind population live in rural areas. Numerous studies from developed and developing countries have demonstrated under-utilization of services in rural areas [36-40]. With the increase in free rural outreach services, it is proposed that a lack of education about common eye diseases and their management is a major contributor to the current roadblock in service utilization. Despite the fact that rural outreach clinics rely on self-presentation, this study showed that awareness of common ocular diseases is significantly lower in this group when compared to the general population in semi urban communities, as well as those who present to urban tertiary eye care centre. This may be attributed to the fact that tools such as print and electronic media, which are the source of information in 52% of cases, are less readily available. Also, the significant disparity in literacy rate between urban (literacy rate of 64%) and rural (literacy rate of 34%) communities may preclude the use of such sources [34]. Further, many patients present despite little awareness of eye diseases because outreach clinics are one of the few free health services available to rural communities and the frequency of service provision is low. However, without adequate awareness and knowledge, the initiative to present to outreach clinics may not translate to acceptance of treatments, such as cataract surgery, or timely presentation to services when they are not free and easily accessible [41]. In fact, one study showed that even when offered free transport and free surgery, the utilization of cataract surgery in parts of rural Nepal was as low as 60% [16]. Therefore, education about the early warning signs of common ocular conditions, the benefits of timely management, and its possible outcomes would be an important addition to current rural outreach services.

Illiteracy is generally associated with lack of disease awareness [13,42,43]. Forty three percent of the Nepalese population is illiterate. Fourteen percent of males and 18% of females have not attended primary school. Fifty four percent of males and 59% females have not attended high school. Female, old age, rural habitation and lower socio-economic class are all risk factors for illiteracy [34]. This is the same groups who carries the highest burden of disease and have the least capacity to access care. Higher level of education beyond simple literacy is associated with better awareness and knowledge of common ocular conditions when compared to those with basic literacy. This may be confounded by other factors such as higher socio-economic background and urban habitation. In summary, it is vital that future health promotion campaigns need to expand beyond traditional means of printed media to make them accessible to illiterate populations who carry high disease burdens.

Multivariate analysis revealed that the awareness of common ocular diseases namely cataract glaucoma, trachoma, night blindness and diabetic retinopathy was significantly associated with level of education (p = <0.05). Similarly, association was found in female gender, age and location of the participants where some diseases had association with common ocular diseases and some were not. Except diabetic retinopathy (p = 0.198), the awareness of common ocular diseases had significant association with female gender (p = <0.05). The awareness of cataract, trachoma, night blindness and diabetic retinopathy had significant association with age (p <0.05). Glaucoma and diabetic retinopathy were significant association with location (p <0.05) but other three ocular conditions like cataract, trachoma and night blindness were not associated with rural outreach camps.

There are some limitations to this study. Firstly, two of the three cohorts (approximate 48.71% of respondents) are already presenting for care either to an urban eye hospital clinic or to a rural outreach clinic, thus, many already demonstrate some awareness of ocular conditions. However, presentation does not necessarily equate to knowledge and awareness, which are essential for identification of early warning signs of disease, compliance to treatment and follow-up. Further, it is common for the majority of the population from a community to present for check-up by the outreach clinics even in the absence of disease or symptom because the service is free and accessible. Therefore, this group offers a relatively representative sample of a rural community. Evidently, there exists much scope for more comprehensive studies on awareness and knowledge of ocular diseases in those from rural communities who have not presented to care, but the recruitment of respondents may be difficult. It is also important to note that while this study examined the relationship between individual determinants of health literacy, it has been alluded to in the discussion that many of these factors co-exist. Therefore, future work is needed to determine the complex interplay between these factors and their collective impact on health literacy and outcome.

## Conclusions

Whilst some progress has been made in the reduction of blindness in Nepal, further improvements necessitate an increase in health literacy of the community. This study shows that even at the sentinel stages of awareness and knowledge, factors such as female gender, old age, illiteracy, lower level of education and rural habitation act as major impediments. Additionally, these factors impacting on health literacy tend to occur simultaneously in the same individuals and producing compounding effects. Strategic actions targeted at these factors are essential in the development of programs that can holistically address all the barriers to the improvement of ocular health.

## Competing interests

The authors declare that they have no competing interests.

## Authors’ contributions

MKS contributed to the design of the study, its implementation and preparation of the manuscript. CWG contributed to the analysis of data, preparation and revision of the manuscript. NM contributed to the collection and analysis of data. RG and SR contributed to the design of the study and preparation of the manuscript. All authors read and approved the final manuscript.

## Pre-publication history

The pre-publication history for this paper can be accessed here:

http://www.biomedcentral.com/1471-2415/14/2/prepub
